# Psoriasis vulgaris arising on a vascular malformation in an 11‐month‐old female child

**DOI:** 10.1002/ccr3.9209

**Published:** 2024-12-12

**Authors:** Jacopo Tartaglia, Ludovica Franceschin, Ina Tudurachi, Christian Ciolfi, Francesca Caroppo, Anna Belloni Fortina

**Affiliations:** ^1^ Dermatology Unit, Department of Medicine (DIMED) University of Padua Padua Italy; ^2^ Pediatric Dermatology Regional Center, Department of Woman's and Children's Health—SDB University of Padua Padua Italy

**Keywords:** dermoscopy, isolated psoriasis, pediatric psoriasis, vascular malformation

## Abstract

While atypical, the development of psoriatic plaques over vascular malformations in children is plausible and should not necessarily prompt clinicians to perform costly or invasive procedures.

## Graphical abstract

A case of psoriasis vulgaris arising on a congenital vascular malformation in an 11‐month‐old female child.

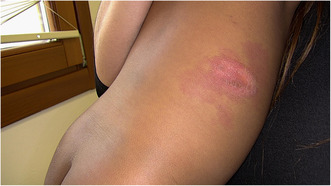

## 1 INTRODUCTION

Psoriasis is a chronic immune‐inflammatory disease that predominantly affects the skin, nails, and joints. Although frequently misidentified, pediatric psoriasis is not uncommon: a recent study has reported a prevalence of psoriasis in the pediatric population of 128 per 100,000, progressively increasing with age.^1^

Notably, childhood‐onset psoriasis accounts for approximately one‐third of all psoriasis cases.^2^

Psoriasis, as a systemic disease, is associated with significant comorbidities, and this relationship extends to the pediatric population.^3^ Previous research indicates that the prevalence of comorbidities in children with psoriasis is approximately 14.4%, which is nearly double the rate observed in children without psoriasis (7.2%).^4^ Children with psoriasis have a two‐ to fourfold increased risk of developing comorbidities such as hyperlipidemia, diabetes mellitus, hypertension, Crohn's disease, and rheumatoid arthritis.^5^, ^6^, ^7^ Psoriatic arthritis, a well‐documented comorbidity in adults, is observed in children with psoriasis, with confirmed prevalence ranging from 6% to 41%.^8^, ^9^

Compared to psoriasis in adults, pediatric psoriasis shows some distinctive features. In particular, pediatric psoriasis is characterized by a more frequent involvement of the face, genital and flexural regions, and a higher prevalence of diaper rash and guttate psoriasis.^2^, ^3^

Capillary malformations are congenital vascular malformations primarily found on the head, neck, and limbs.^10^ These chronic malformations, although not typically posing health risks, may impact the quality of life of children due to aesthetic concerns.^11^ Histologically, they consist of ectatic capillary‐to‐venular‐size vessels in the upper dermis.^10^ Although they can be emptied of blood and blanched upon compression, capillary malformations do not fade and remain permanent throughout life.^12^

Currently, it is unknown whether vascular malformations may serve as a preferred site for inflammatory dermatoses. In this article, we present the case of psoriasis vulgaris arising on a congenital vascular malformation.

## 2 CASE HISTORY

**FIGURE 1:**
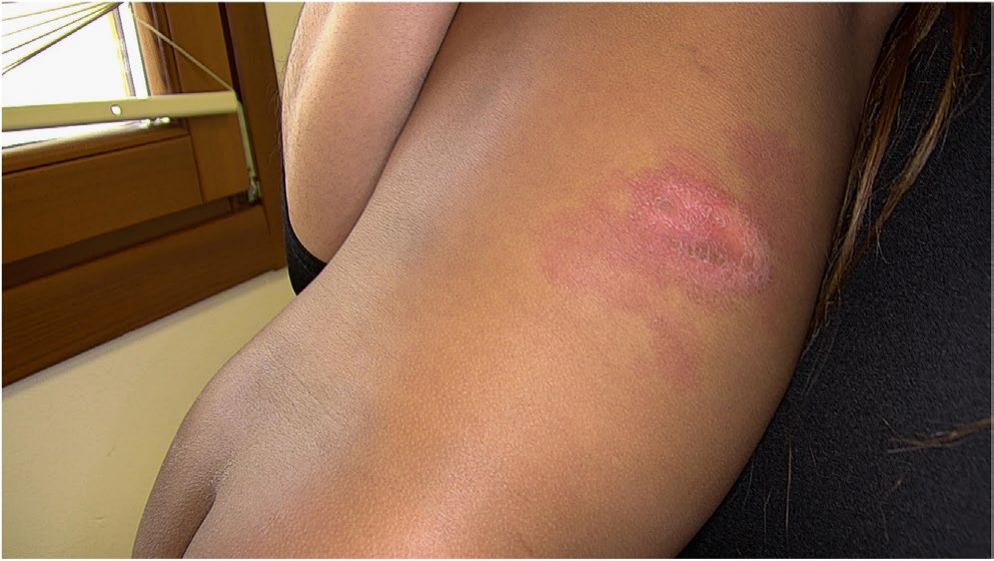
A well‐defined erythematous scaly plaque within a large capillary malformation (maximum diameter of 6 × 4 cm) in an 11‐month‐old girl.

**FIGURE 2:**
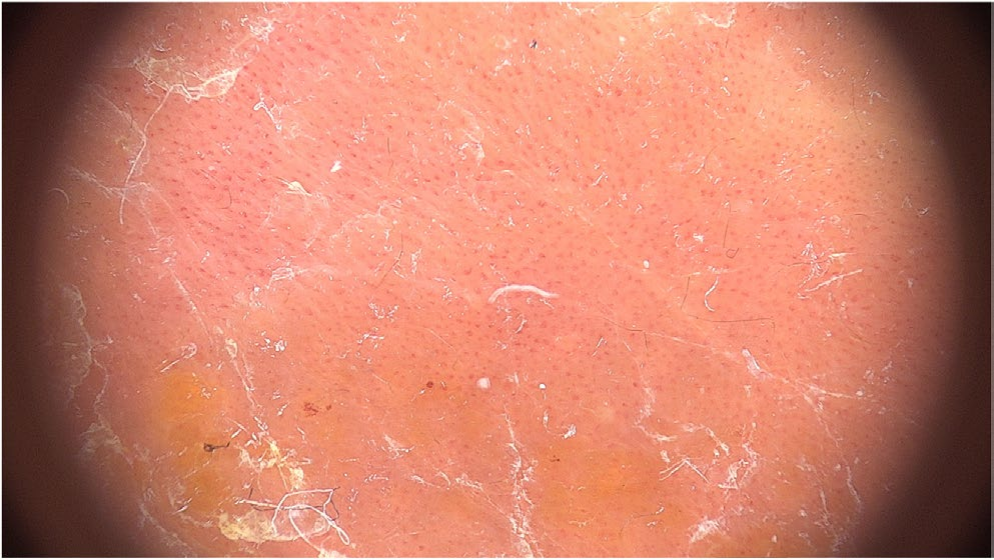
Dermoscopy reveals evenly distributed red dots and globules over an erythematous background, accompanied by white scales.

An 11‐month‐old female child was referred to our Pediatric Dermatology Unit for the evaluation of a large vascular malformation (maximum diameter of 6 × 4 cm), which was present at birth, and a rash in the diaper area, accompanied by erythema and desquamation of the elbows and retroauricular regions. A clinical diagnosis of capillary malformation and of psoriasis vulgaris with concomitant inverse psoriasis was established. The patient was prescribed with topical treatment consisting of emollients, low‐potency steroids, and topical pimecrolimus, with complete resolution of psoriatic lesions within a few weeks. However, approximately 9 months after the initial diagnosis the patient developed an erythematous scaly plaque on the right hemithorax, over the preexisting vascular malformation (Figure 1); the Auspitz sign (pinpoint bleeding after scraping off the lesions) was observed. Dermoscopy revealed regularly distributed red dots and globules over a reddish background, along with white scales (Figure 2). The clinical and dermoscopic features were suggestive of plaque psoriasis occurring on a vascular malformation.

## 3 METHODS

The dermoscopic examination aided in the differentiation of active psoriatic plaques within the context of a large capillary malformation. Indeed, the dermoscopic features of a capillary malformation typically consist of vascular features occupying more than 75% of the lesion.^13^ In our case, the presence of clinical and dermoscopic signs suggestive of plaque psoriasis interrupting the vascular features present throughout the lesion allowed for the diagnosis. The main differential diagnosis was eczema on vascular malformation (configuring the Meyerson phenomenon). Nonetheless, the complete absence of itchiness and dermoscopic criteria suggestive of eczema (yellow scales and serocrusts, dotted vessels distributed in clusters),^14^ together with the presence of psoriatic lesions elsewhere on the body, supports the clinical diagnosis of psoriasis vulgaris over dermatitis.

## 4 CONCLUSION AND RESULTS

A course of topical low‐potency steroids was prescribed with complete resolution. Additionally, a maintenance therapy with topical pimecrolimus was initiated. At the 1‐year follow‐up, the patient was in complete remission with the use of topical pimecrolimus twice weekly.

## 5 DISCUSSION

There are no data available about psoriasis vulgaris developing on vascular malformations. However, there have been reported cases of eczematous lesions appearing on these malformations, configuring the “Meyerson phenomenon.” Meyerson phenomenon has been extensively studied in relation to melanocytic lesions, but its occurrence over vascular anomalies is rarely observed.^15^ The factors underlying this association are unknown, still a few hypotheses can be made. First, it is possible that vascular stasis and increased blood flow in the affected area contributed to the accumulation of pro‐inflammatory cytokines.^16^

This could potentially create a local environment conducive to the development of inflammatory skin conditions, including both eczematous lesions and psoriasis. In the context of eczematous dermatitis, the inflammatory milieu is often dominated by a Th2‐driven immune response, characterized by cytokines such as IL‐4, IL‐5, and IL‐13. These cytokines, along with chemokines like CCL17 and CCL22, play a crucial role in recruiting and activating eosinophils and other immune cells, leading to the chronic inflammation and itchiness typical of eczema.^17^

On the other hand, psoriasis is primarily driven by a different set of inflammatory pathways. The IL‐23/IL‐17 axis is particularly significant in the pathogenesis of psoriasis, with IL‐23 promoting the differentiation and expansion of Th17 cells, which in turn produce IL‐17A, IL‐17F, and IL‐22.^18^ These cytokines are potent mediators of keratinocyte activation and proliferation, contributing to the characteristic psoriatic plaques. Additionally, TNF‐α is a key pro‐inflammatory cytokine involved in both the initiation and perpetuation of psoriatic lesions, acting synergistically with other cytokines to amplify the inflammatory response.^19^

Second, the manifestation of psoriasis in this particular site may be purely coincidental, considering that psoriasis can occur anywhere on the body. Lastly, the occurrence of psoriasis in this unique site could be attributed to the Koebner phenomenon, as mechanical stress on the area might have triggered its development. In conclusion, we believe that the development of psoriatic plaques within vascular malformations is possible, although it cannot be considered typical. While the underlying factors are not known, it is important for clinicians to be aware of this possibility to avoid diagnostic errors and unnecessary invasive tests. In addition, the prompt response to topical therapy, including topical steroids for the acute phase and topical calcineurin inhibitors for maintenance, was sufficient to induce complete remission. This suggests that psoriasis developing over vascular malformations does not necessarily require more aggressive or systemic therapies but can be managed similarly to psoriasis occurring elsewhere on the body with equivalent PASI scores.

## AUTHOR CONTRIBUTIONS

**Jacopo Tartaglia:** Conceptualization; visualization; writing – original draft; writing – review and editing. **Ludovica Franceschin:** Conceptualization; writing – original draft; writing – review and editing. **Ina Tudurachi:** Conceptualization; writing – original draft; writing – review and editing. **Christian Ciolfi:** Formal analysis; supervision; writing – review and editing. **Francesca Caroppo:** Methodology; writing – review and editing. **Anna Belloni Fortina:** Conceptualization; validation; writing – review and editing.

## FUNDING INFORMATION

None to declare.

## CONFLICT OF INTEREST STATEMENT

None to declare.

## CONSENT

Parents gave verbal and written informed consent to publication of case details. Written informed consent was obtained from the patient to publish this report in accordance with the journal's patient consent policy.

## Data Availability

Data available on request from the authors.
